# Influences of Psychological Traits and PROP Taster Status on Familiarity with and Choice of Phenol-Rich Foods and Beverages

**DOI:** 10.3390/nu11061329

**Published:** 2019-06-13

**Authors:** Alessandra De Toffoli, Sara Spinelli, Erminio Monteleone, Elena Arena, Rossella Di Monaco, Isabella Endrizzi, Tullia Gallina Toschi, Monica Laureati, Fabio Napolitano, Luisa Torri, Caterina Dinnella

**Affiliations:** 1 Department of Agricultural, Food, Environment and Forestry Science and Technology (DAGRI), University of Florence, 50144 Florence, Italy; alessandra.detoffoli@unifi.it (A.D.T.); sara.spinelli@unifi.it (S.S.); caterina.dinnella@unifi.it (C.D.); 2 Department of Agriculture, Food and Environment (Di3A), University of Catania, 95123 Catania, Italy; earena@unict.it; 3 Department of Agriculture Sciences, University of Naples Federico II, 80055 Naples, Italy; dimonaco@unina.it; 4 Fondazione Edmund Mach, 38010 San Michele all’Adige (TN), Italy; isabella.endrizzi@fmach.it; 5 Department of Agricultural and Food Sciences (DISTAL), University of Bologna—Alma Mater Studiorum, 40127 Bologna, Italy; tullia.gallinatoschi@unibo.it; 6 Department of Food, Environmental and Nutritional Sciences (DeFENS), University of Milan, 20133 Milan, Italy; monica.laureati@unimi.it; 7 School of Agricultural, Forestry and Environmental Sciences (SAFE), University of Basilicata, 85100 Basilicata, Italy; fabio.napolitano@unibas.it; 8 University of Gastronomic Sciences, 12042 Pollenzo (CN), Italy; l.torri@unisg.it

**Keywords:** choice, familiarity, PROP, food neophobia, sensitivity to disgust, sensitivity to punishment, vegetables, caffeinated beverages, bitterness, astringency

## Abstract

Plant phenolics are powerful antioxidants and free radical scavengers that can contribute to the healthy functional properties of plant-based food and beverages. Thus, dietary behaviours rich in plant-based food and beverages are encouraged. However, it is well-known that the bitter taste and other low-appealing sensory properties that characterize vegetables and some other plant-based foods act as an innate barrier for their acceptance. The aim of this study was to investigate the influence of psychological traits and PROP status (the responsiveness to bitter taste of 6-n- propylthiouracil) on the choice of and familiarity with phenol-rich vegetables and beverages varying in recalled level of bitterness and astringency. Study 1 aimed at assessing the variations of the sensory properties of vegetable and coffee/tea items with two check-all-that-apply (CATA) questionnaires (*n* = 201 and *n* = 188 individuals, respectively). Study 2 aimed at investigating how sensitivity to punishment, to reward, and to disgust, food neophobia, private body consciousness, alexithymia, and PROP responsiveness affect choice and familiarity with phenol-rich foods (*n* = 1200 individuals). A Choice Index was calculated for vegetables (CV) and coffee/tea (CC) as a mean of the choices of the more bitter/astringent option of the pairs and four Familiarity Indices were computed for vegetables (FV) and coffee/tea (FC), higher (+) or lower (-) in bitterness and astringency. Subjects higher in food neophobia, sensitivity to punishment or sensitivity to disgust reported significantly lower choice indices than individuals lower in these traits, meaning that they systematically opted for the least bitter/astringent option within the pairs. Familiarity with vegetables was lower in individuals high in sensitivity to punishment, in food neophobia and in alexithymia, irrespective of their sensory properties. The Familiarity Index with coffee/tea characterized by higher bitterness and astringency was lower in individuals high in food neophobia, sensitivity to disgust, and alexithymia. No significant effect of PROP was found on any indices. The proposed approach based on product grouping according to differences in bitterness and astringency allowed the investigation of the role of individual differences in chemosensory perception and of psychological traits as modulators of phenol-rich foods preference and consumption.

## 1. Introduction

Diets rich in plant-based food and beverages are encouraged, given general agreement on their positive health outcomes. Meta-analyses of the effects of such foods indicate that a reduced risk of coronary heart disease, stroke, and diabetes are associated with a regular intake of non-starchy vegetables and moderate consumption of tea and coffee [1].

Plant phenolics are powerful antioxidants and free radical scavengers that can contribute to the healthy functional properties of plant-based food and beverages [2]. However, phenol compounds from vegetable sources are characterized by bitterness, astringency, and pungency [3,4,5], sensations that may limit food acceptability [6,7]. Human beings, long sensitized to the bitter taste of plant toxins, consider excessive bitterness the principal reason for food rejection [8]. The tactile sensation of astringency discourages animals from ingesting foods too high in tannins, thus protecting them from the tannin’s potential harmful anti-nutritional effects [9]. A high intensity of perceived astringency negatively impacts the acceptance for high phenol containing foods [3]. The high phenol binding proteins from parotid glands exert a protective role against dietary phenols, and astringency arises from phenol interactions with the adsorbed glycoprotein layer, with the consequent oral cavity delubrication [10,11].

Sensory properties drive liking for vegetables [12], and it is well-known that bitterness and other unpalatable sensory properties may act as a barrier for vegetable acceptance [8,9,13,14]. Moreover, while bitterness and astringency are important qualities in tea and coffee, and may contribute to consumer appreciation of these products [15,16], in actual consumption conditions, masking ingredients (sweeteners, milk) are often used to modify these sensations to levels compatible with individual preferences [17]. 

Healthy individuals substantially differ in chemosensory perception, and such variability has been extensively studied in recent years. Most notably, the inherited capacity to perceive the bitterness of propylthiouracil (PROP) is considered a reliable broad marker for individual differences in taste responsiveness that may influence food preferences and eating behaviour [18]. The effect of the PROP phenotype (PROP bitterness ratings on the generalized Labeled Magnitude *Scale* (gLMS): ≤17, non-taster (NT); 18–52, medium taster (MT); and ≥53, supertaster (ST), according to Hayes et al. and Fischer et al. [19,20]) on the intake and preference of bitter foods and beverages has been examined in several studies, with mixed results, mainly because demographics, genetics, and other environmental factors may influence both phenotypic responses to oral stimulation and affective response to food [21,22]. Those who are insensitive to PROP bitterness (non-tasters) were found to consume more vegetables and more bitter vegetables than the other taster phenotypes, PROP medium-tasters and super-tasters [23,24]. The super-taster PROP phenotype was associated with a lower preference for bitter vegetables [25]. On the other hand, no differences between PROP phenotypes were found in preferences for plant-based bitter foods [26] or for actual vegetable intake in children [27,28,29]. PROP supertasters gave higher bitterness, sourness, and astringency ratings for coffee, but these did not significantly affect liking [17] or consumption [30]. In general, these results are inconsistent and the causal models envisaging straight associations of variations in taste abilities with food perception and choice show a weak predictive power. 

Recent studies have shown that personality has a hugely important role in preferences and choices and, in some cases, in determining sensory responses to foods. One such key personality variable is the trait of food neophobia (FN), originally defined as the reluctance to try or eat unfamiliar foods. High levels of food neophobia have been associated with reduced preference and intake for many food products belonging to different categories, including fruits and vegetables, in adults [31,32] and children [33]. In particular, food neophobia was found to affect the liking of foods and beverages characterized by high intensities of bitterness, astringency, sourness, and pungency. Those high in food neophobia (neophobics) reported liking such vegetables, beverages, fruits, and spicy foods less than those low in food neophobia (neophilics). Conversely, few differences between food neophobia groups were found for the liking of bland vegetables and beverages, or for sweets and desserts [32,34]. Neophobics perceive pungency and astringency in food products as more intense, and like the most pungent and astringent samples less than neophilics [34,35]. 

Other personality traits have been found to be associated with lower preferences for pungent foods. Individuals highly sensitive to visceral disgust (disgust related to rotten food, vermin, and body fluids) [36,37] find pungent foods more intense and like and choose them less [35]. Two other personality traits, sensitivity to punishment and sensitivity to reward, describe individual differences in reactivity and responsivity to the behavioural inhibition and activation systems, respectively [38]. Sensitivity to punishment was found to be negatively associated with liking of spicy foods [39] and pungent food choice in females [35]. Sensitivity to reward was found to be positively associated with chili intake, liking of spicy foods, and choice of pungent foods [35,39,40]. Recent studies have also highlighted an association between sensitivity to reward and unhealthier food behaviours, such as a preference for sweet and fatty foods, higher fat intake, higher alcohol consumption, and smoking frequency [41,42,43]. Alexithymia, defined as the inability of individuals to identify and name their emotional states [44], was found to be associated with food preferences, with high alexithymia associated with a liking for alcohol, sweets, and fats/meats, and lower alexithymia with a liking for vegetables, condiments, and strong cheeses [45].

The complexity of these factors and the sometimes mixed reports on their effects indicate that the interplay of several dimensions, such as gender, age, personality traits, and taste responsiveness, influence choice and intake of foods and beverages. In addition, food products are selected based on culture, which means that some products are far more contextually appropriate and/or familiar than others. While a positive relationship between familiarity and choice can be expected, the strength of this relationship is unclear. Many contextual situational factors may play a role in choice, while familiarity covers both features of frequency of consumption (occasional and regular) and levels of knowledge (from product name to product taste) that are less affected by contextual factors (see, for example, the scale developed by Tuorila and colleagues [46]). In addition, it is not known if, or in what way, the relationship between choice and familiarity is affected by personality traits or taste responsiveness. Although some studies have investigated how taste responsiveness affects food familiarity or food choice, the literature on the role of psychological traits is quite limited, and the relationships between these variables remain little explored [35]. Exploring the factors that influence choice of and familiarity with phenol-rich foods and beverages is of interest to better understand food behaviour and to shed light on the role of personality traits and taste responsiveness as barriers to heathy eating.

The grouping of food and beverages based on their overall sensory characteristics has already been used to explore individual differences in preferences and consumption. PROP status only marginally affects the preference expressed for specific foods selected to represent sensations generally disliked by PROP supertasters, such as bitterness and pungency [26]. Food neophobia level significantly influenced preference for and familiarity with food and beverages categorized as “mild” and “strong” flavors [34]. Grouping vegetables as having low and high appeal was used to investigate demographic and attitudinal variables affecting vegetable consumption in European adolescents [14]. Existing data from sensory evaluations of trained and untrained assessors, as well as the chemical composition, were the criteria generally used for grouping the foods [12,14,47,48,49,50,51]. 

In the present study, an original approach to phenol-rich product grouping based on differences in bitterness and astringency is proposed. This approach was used to investigate the influence of individual variation in psychological traits and PROP status on choice of and familiarity with phenol-rich vegetables and beverages, varying in recalled levels of bitterness and astringency. Furthermore, the relationship between familiarity with and choice of phenol-rich vegetables and beverages with a high recalled level of bitterness and astringency as a function of personality traits and PROP status was investigated.

## 2. Materials and Methods

The experimental plan consisted of two independent studies: one preliminary study and one main study, conducted with two different subject groups. The preliminary study was conducted in order to validate the differences in expected level of bitterness and astringency within each pair included in the vegetable choice questionnaire (V-IT-FCQ) and coffee/tea choice questionnaire (C-IT-FCQ) used in the main study. The main study aimed at investigating how PROP responsiveness and psychological traits affect familiarity with, and choice of, vegetables and coffee/tea, presented in pairs with two options with different levels of bitterness and astringency. The studies were conducted in agreement with the Italian ethical requirements on research activities and personal data protection (D.L. 30.6.03 n. 196) and the respondents gave their written informed consent at the beginning of the study. The protocol of the studies was approved by the Ethics Committee of Trieste University. The respondents gave their written informed consent at the beginning of the test, according to the principles of the Declaration of Helsinki.

### 2.1. Participants

Participants were recruited on a national basis by means of announcements published on social networks (Facebook), articles published in national newspapers, and in magazines. Furthermore, each research unit recruited subjects locally by means of social networks, mailing lists, pamphlet distribution, and word of mouth. The exclusion criteria were pregnancy and not having lived in Italy for at least 20 years.

#### 2.1.1. Preliminary Study—Validation of the Differences in Bitterness and Astringency within Pairs of the Choice Questionnaires used in the Main Study

Subjects completed an online questionnaire aimed at measuring the sensory response (bitterness and astringency) to vegetables (201 subjects: 77.7% females; age range 18–70; mean age 40.3 ± SD 14.1) and coffee/tea (188 subjects: 75.4% females; age range 19–68; mean age 40.1 ± SD 14.3) products (presented with names) selected for the questionnaires used in the main study (§ 2.1.2). 

#### 2.1.2. Large Scale Data Collection 

Data were collected on 1200 Italian subjects (58% females; age range 18–60 years; male mean age 35.9 years ± SD 12.8; female mean age: 35.2 years ± SD 12.9) on a national basis. In order to explore possible age-related differences, subjects were divided into three age groups: 18–30 years (45.6%), 31–45 years (28.0%), 46–60 years (26.4%).

### 2.2. Procedure

#### 2.2.1. Preliminary Study—Validation of the Differences in Bitterness and Astringency within Pairs of the Choice Questionnaires

Two check-all-that-apply (CATA) questionnaires [52] with forced choice (yes/no) were developed to describe the sensory properties of items to be included in the vegetable food choice questionnaire (V-IT-FCQ) and coffee/tea choice questionnaire (C-IT-FCQ) used in the main study. The vegetable CATA questionnaire included fourteen items: “pumpkin risotto”, “risotto with radicchio”, “lettuce and valerian salad” (*Valerianella locusta,* also known as corn salad or mâche), “radicchio and rocket salad”, “green salad”, “bean sprout salad”, “chard”, “chicory”, “zucchini”, “asparagus”, “carrots”, “cauliflowers”, “cucumber”, and “radish”. The coffee/tea CATA questionnaire included coffee and tea items with/without ingredients (milk and sugar) masking the perception of bitterness and astringency. The coffee/tea CATA questionnaire included six items: “coffee with sugar”; “coffee without sugar”; “tea with sugar”; “tea without sugar”, “macchiato”, and “cappuccino”. The list of sensory properties included 19 and 13 descriptors in the vegetable and coffee/tea questionnaires, respectively, but in the present paper only bitterness and astringency were considered. Both the products and the sensory properties were presented using words in a randomized order. The participants filled in the questionnaire online. The online platform SurveyGizmo (surveygizmo.eu) was used for data collection. 

#### 2.2.2. Large Scale Data Collection

Participants were asked to fill in an online questionnaire, and they then attended a session at the laboratory. Socio-demographic (gender, age, education) information and familiarity with foods were collected through online questionnaires before the test sessions. In the lab session, participants were asked to fill in a set of questionnaires to measure personality and psychological traits and to complete the choice questionnaires. PROP responsiveness was also measured. The study included sensory tests, questionnaires, and the collection of other data (see Monteleone et al., [53] for a complete overview of data collection), but only a selection of variables are presented here.

##### Psychological Traits

Sensitivity to punishment (SP) and sensitivity to reward (SR), related to responsiveness of behavioural inhibition and activation systems, were quantified using the sensitivity to punishment and sensitivity to reward questionnaire (SPSRQ) questionnaire developed by Torrubia, Ávila, Moltó, and Caseras [54]. Items 4, 8, 16, 25, 32, 34, and 36 were discarded based on the validation of the questionnaire in Italian (see Spinelli et al 2018 [36]). The sensitivity to punishment and sensitivity to reward scales were scored with a yes/no format. For each subject, sensitivity to punishment and sensitivity to reward scores were computed by summing up the yes answers (SP score range 0–23; SR score range 0–18), so that a higher score indicated a higher sensitivity to punishment and to reward.

Food neophobia (FN), defined as the reluctance to try and eat unfamiliar foods, was quantified using the 10-statement scale developed by Pliner and Hobden [55] and validated in Italian by Laureati and colleagues [34]. Individual food neophobia scores were computed as the sum of ratings given to the 10 statements, after reversing the neophilic items (using a seven point Likert scale: disagree strongly/agree strongly). The scores ranged from 10 to 70, with higher scores corresponding to higher food neophobia.

Sensitivity to disgust (DS), defined as the responsivity to core-visceral disgust (rotten food, vermin, body fluids), was quantified using the eight-item short form of the disgust sensitivity scale developed by Inbar, Pizarro, and Bloom [56] and validated in Italian by Spinelli and colleagues [35]. The scale includes two subscales, each presented with a specific scale ranging from 1 = strongly disagree (very untrue about me) to 5 = strongly agree (very true about me) (subscale 1) and from 1 = not at all disgusting to 5 = extremely disgusting (subscale 2). The individual scores ranged from 5 to 40, with higher scores reflecting a higher sensitivity to disgust. 

Private body consciousness (PBC), defined as the disposition to focus on internal bodily sensations (awareness of internal sensations), was quantified using the five-item instrument developed by Miller, Murphy, and Buss [57]. The individual score was computed as the sum of the ratings given for the five statements (using a five-point scale: extremely uncharacteristic/extremely characteristic). The scores ranged from 5 to 25, with higher scores reflecting higher private body consciousness levels.

Alexithymia (TAS), defined as a specific disturbance in psychic functioning, characterized by difficulties in the capacity to verbalize affect and to elaborate fantasies, was quantified using the Toronto Alexithymia Scale (TAS) developed by Parker, Bagby, Taylor, Endler, and Schmitz [58] and validated in Italian by Bressi and colleagues [59]. The individual alexithymia total score was computed as the sum of ratings given to the 20 statements (using a five-point Likert scale: disagree strongly/agree strongly). The alexithymia total scores ranged from 20 to 100, with a higher score indicating a greater level of alexithymia.

##### PROP Phenotyping

PROP taster status was assessed using a 3.2 mM PROP solution, prepared by dissolving 0.545 g/L of 6-n-propyl-2-thiouracil (European Pharmacopoeia Reference Standard, Sigma Aldrich, Milano, Italy) in deionized water [60]. Subjects were presented with two identical 10 mL samples, each coded with a three-digit code. Subjects were instructed to hold each sample in their mouth for 10 s, then to expectorate, wait 20 s, and evaluate the intensity of bitterness using the general label magnitude scale (gLMS; 0 = no sensation–100 = the strongest imaginable sensation of any kind) [61]. Verbal instructions were given that the top of the scale represented the most intense sensation that subjects could ever imagine experiencing. To ensure appropriate use of this scale, practise using a variety of remembered sensations from different modalities, including loudness, oral pain/irritation, and tastes, was provided. Subjects had a 90 s break to control for carry-over effects after the first sample evaluation. During the break, subjects adopted a washing procedure to rinse their mouth with distilled water for 30 s, ate some plain crackers for 30 s, and finally rinsed with water for a further 30 s before they evaluated the second PROP sample [5]. PROP taster status was based on the average rating of the two replicates and groupings were based on previously published cut-offs [19,20]: PROP non-tasters (NT) ≤17 (*n* = 274); PROP medium tasters (MT), 18–52 (*n* = 505); and PROP supertasters (ST) ≥53 (*n* = 421) on the gLMS.

##### Choice of and Familiarity with Vegetable and Coffee/Tea items

The choice of phenol-rich vegetables and coffee/tea between pairs of two food items characterized by different levels of bitterness and astringency was assessed with the V-IT-FCQ and C-IT- FCQ (Table 1). Vegetable and coffee/tea pairs in the choice questionnaires were selected so that the options in each pair significantly differed for bitterness and astringency, based on the results of the preliminary CATA study. V-IT FCQ consisted of seven pairs of vegetables, selected to represent possible options for the same main dish (risotto with different condiments: pumpkin or zucchini) and for similar side dishes consisting of raw (leafy/green salads: lettuce and valerian or radicchio and rockets; green salad or bean sprouts; salad ingredients: cucumbers or radishes) or cooked (leafy green: chard or chicory; others: zucchini or asparagus; carrot or cauliflower) vegetables. Similarly, coffee and tea options were selected to represent possible alternatives of the same hot beverage, including or excluding ingredients masking the perception of bitterness and astringency (i.e., milk and sweeteners). 

For each pair, participants were asked to indicate which food they would ideally choose, pointing out that the answer would describe not what they usually choose but rather what they would like to choose in a situation of absence of restrictions (e.g., due to health or weight concerns). The choice for vegetables was asked in the context of a main meal and the choice for coffee/tea was asked in the context of breakfast. Options within the pairs were coded as “0” for the lowest level of bitterness and astringency and “1” for the highest level of bitterness and astringency. Here, for each subject, a choice index was calculated for vegetables (CV) and coffee/tea (CC) as a mean of the choices of the more bitter/astringent option (range from 0 to 1). Transformation in continuous variables of the binary data has been proposed in order to simplify analysis and use standard statistical methods frequently used for sensory data [62,63]. The approach for the calculation of a choice index as a sum of the options 1 (within the pairs) was already used in Spinelli et al. [35]. 

Familiarity with vegetables and coffee/tea items was assessed by a five-point labelled scale (1 = I do not recognize it; 2 = I recognize it, but I have never tasted it; 3 = I have tasted it, but I don’t eat it; 4 = I occasionally eat it; 5 = I regularly eat it) developed by Tuorila and colleagues [46]. Two indices of familiarity with vegetables and coffee/tea higher in bitterness and astringency (+) were obtained by the sum of ratings of familiarity with the items that, within each pair, were higher in these sensations, based on the results of the preliminary study: FV+: risotto with radicchio, radicchio and rocket salad, bean sprout salad, chicory, asparagus, cauliflower, radish; ranging from 7 to 35; FC+: coffee and tea without sugar; ranging from 2 to 10. Two indices of familiarity with vegetables and coffee/tea lower in bitterness and astringency, respectively, were obtained by the sum of ratings of familiarity with the items that, within each pair, showed a lower level of bitterness and astringency (-), based on the results of the preliminary study: FV-: pumpkin risotto, lettuce and valerian salad, chard, zucchini, carrots, cucumber; ranging from 6 to 30; FC-: coffee and tea with sugar; ranging from 2 to 10. 

The presentation order of the food items in the familiarity and choice questionnaires was randomized across participants. 

### 2.3. Data Analysis

#### 2.3.1. Preliminary study—Validation of the Differences in Bitterness and Astringency within Pairs of the Choice Questionnaires

Cochran Q-tests were performed to assess the differences between the frequency of selection of bitterness and astringency within the pairs of the V-IT-FCQ and C-IT-FCQ. Post-hoc pairwise comparisons were calculated using the McNemar procedure and the level of significance was set at 5% [43,52].

#### 2.3.2. Large Scale Study

Cronbach’s α was computed to check for the internal reliability of each psychological trait questionnaire. Two-way ANOVA models were used to determine the main effects of gender (males; females) and age class (18–30; 31–45; 46–60) and their interactions on psychological trait scores and on PROP bitterness intensity. Three-way ANOVA models were used to test the effects of gender, age, and psychological trait level (low, medium, and high) and PROP status (NT, MT, and ST) and their interactions on choice (CV and CC) and familiarity (FV+, FV-, FC+, FC-) indices. 

The robustness of the ANOVA models was verified; the residuals of each ANOVA model were inspected for normality by histograms and Q–Q plots and for heteroscedasticity using Levene’s test. A *p*-value of 0.05 was considered the threshold for statistical significance and post-hoc using the Bonferroni test adjusted for multiple comparisons were used. Pearson’s correlation coefficients were computed to explore the association between familiarity and choice (FV+ and CV; and FC+ and CC, respectively) in subject groups with different levels of expression of psychological traits (L, M, and H) and PROP status (NT, MT, and ST). A *p*-value of 0.05 was considered the threshold for statistical significance. Fisher’s r to z transformation was used on the correlation coefficient to assess the significance of the differences (*p*-value of 0.05).

The XLSTAT statistical software package version 19.02 (Addinsoft) was used for data analysis. 

## 3. Results

### 3.1. Preliminary Study—Validation of the Differences in Bitterness and Astringency within Pairs of the Choice Questionnaires

Significant differences were found between the items of each pair belonging to the vegetable choice questionnaire (V-IT-FCQ) and to the coffee/tea choice questionnaire (C-IT-FCQ) in both bitterness and astringency frequency of selection, with the exception of green salad/bean sprout salad in bitterness (*p* = 0.262) and carrots and cauliflower in astringency (*p* = 0.827) (Table 2). 

### 3.2. Large Study on Familiarity with and Choice of Phenol-Rich Foods and Beverages 

#### 3.2.1. Personality Trait Questionnaires

The internal reliability of the questionnaires measuring psychological traits was satisfactory, with Cronbach’s alpha ranging from 0.86 to 0.70 (Table 3). Based on the percentile limits, the population was grouped into Low-L (1° quartile), Medium-M (interquartile), and High-H (3° quartile) levels of expression of each trait (Table 3). 

Both gender and age affected individual variation in personality traits (Table 4). A significant gender effect was found for private body consciousness, sensitivity to punishment, sensitivity to reward, and sensitivity to disgust. Females were significantly higher in private body consciousness, sensitivity to punishment, and sensitivity to disgust than males, while males were more sensitive to reward. A significant effect of age was found for sensitivity to punishment, sensitivity to reward, sensitivity to disgust, alexithymia, and food neophobia. Sensitivity to punishment, sensitivity to reward, and alexithymia decreased with age, while food neophobia and sensitivity to disgust increased with age. The effect was further characterized by an interaction in the case of gender with private body consciousness: a decrease in private body consciousness with age was found in males, but not in females.

#### 3.2.2. PROP Responsiveness

Effects of both gender and age were found on responsiveness to PROP (Table 4). The effects were further characterized by an interaction with gender, in that females were more responsive to PROP. PROP responsiveness decreased from the age class 18–30 to 31–45 and then remained stable in females, while a decrease in PROP responsiveness in males was reported in the age class 46–60. 

#### 3.2.3. Vegetable Choice Index (CV) and Coffee/Tea Choice Index (CC)

The effects of individual variation in psychological traits and PROP status, gender, age, and their interactions on choice indices are reported in Table 5. 

A significant effect of both gender and age was found for the vegetable choice index in each ANOVA model. The coffee/tea choice index was significantly affected by age only in the food neophobia and sensitivity to disgust models, while no effect of gender on the coffee/tea choice index was reported. These effects were not further characterized by an interaction between gender and age. The vegetable choice index was higher in males and increased with age. When the effect was found to be significant, the coffee/tea choice index increased with age. 

**Figure 1:**
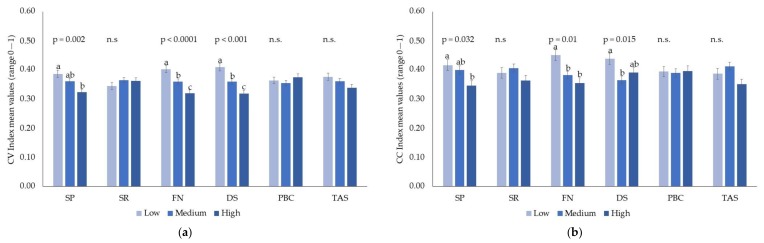
(**a**) Effects of psychological traits (sensitivity to punishment, SP; sensitivity to reward, SR; food neophobia, FN; sensitivity to disgust, DS; private body consciousness, PBC; and alexithymia, TAS) on the choice index for vegetables (CV Index). (**b**) Effects of psychological traits (sensitivity to punishment, SP; sensitivity to reward, SR; food neophobia, FN; sensitivity to disgust, DS; private body consciousness, PBC; and alexithymia, TAS) on the choice index for coffee/tea (CC). Different letters represent significantly different values (*p* ≤ 0.05). n.s.= non-significant (*p* > 0.05).

The effect of food neophobia, sensitivity to punishment, and sensitivity to disgust was significant for both the vegetable choice index and coffee/tea choice index. These effects were not further characterized by interactions with age and gender. Individuals who scored higher in food neophobia, sensitivity to punishment, or sensitivity to disgust reported significantly lower choice indices than individuals low in these traits, meaning that they systematically opted for the least bitter/astringent option within the pairs (Figure 1a–b).

A significant interaction was found for alexithymia (TAS) and gender (coffee/tea choice index), but no significant difference was found in a Bonferroni pairwise comparison. A significant interaction was found for private body consciousness (PBC) and gender (vegetable choice index), with males medium and high in private body consciousness reporting a higher choice index than females medium and high in private body consciousness. 

PROP responsiveness. No effect of PROP responsiveness was found on either choice index. 

#### 3.2.4. Familiarity with Vegetables (FV+ and FV-) 

Individual variation in psychological traits significantly affected familiarity with vegetables in the case of sensitivity to punishment (F = 9.6; *p* < 0.0001), food neophobia (F = 30.1; *p* < 0.0001), disgust sensitivity (F = 7.8 *p* = 0.0004), and alexithymia (F = 8; *p* = 0.0003). Higher levels in these traits corresponded to a lower familiarity with vegetables. This was further investigated, considering the vegetable groups varying in bitter and astringency. Table 5 reports the effects of individual variation in psychological traits and PROP status, gender, age, and their interactions on familiarity indices with vegetables high (+) and low (-) in bitterness and astringency. 

A significant effect for both age and gender was found on the familiarity index for vegetables higher in bitterness and astringency and the familiarity index for vegetables lower in bitterness and astringency in each ANOVA model, with the only exception being gender in the model with food neophobia. These effects were not further characterized by an interaction (gender and age). Females were more familiar with vegetables irrespective to their bitterness and astringency level. Both vegetable familiarity indices increased with age.

**Figure 2:**
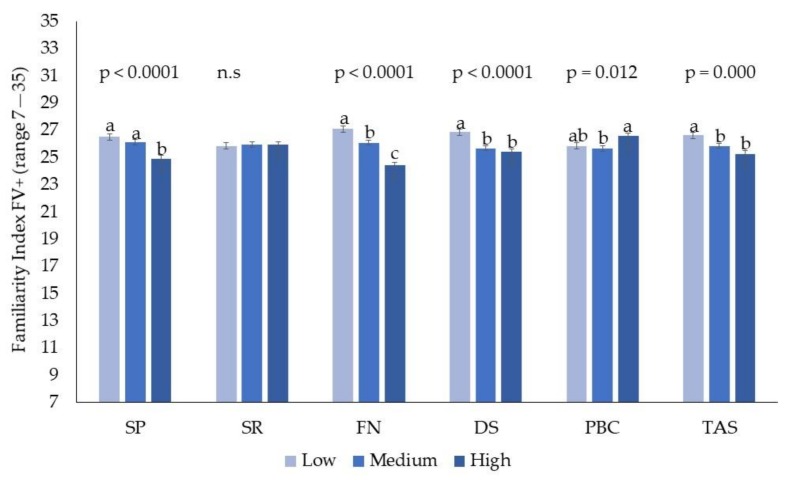
Effect of psychological traits (sensitivity to punishment, SP; sensitivity to reward, SR; food neophobia, FN; sensitivity to disgust, DS; private body consciousness, PBC; and alexithymia, TAS) on the familiarity index with vegetables higher in bitter and astringency (FV+). Different letters represent significant different values (*p* ≤ 0.05). n.s.= non-significant (*p* > 0.05).

A significant effect for food neophobia, alexithymia, and sensitivity to punishment was found on both indices, while a significant effect for private body consciousness and sensitivity to disgust was found only on the familiarity index with vegetables higher in bitterness and astringency. These effects were not further characterized by an interaction with age or gender. Both familiarity indices were lower in neophobics, in individuals higher in sensitivity to punishment and higher in alexithymia. The familiarity index with vegetables characterized by high unappealing sensations was lower in individuals higher in sensitivity to disgust. For private body consciousness, the post hoc test did not show significant differences between individuals high and low in this trait. The effect of individual variation in psychological traits on the familiarity index for vegetables high in bitterness and astringency is reported in Figure 2. 

No effect of PROP responsiveness was found on either index, while a significant interaction between PROP and gender was observed on the familiarity index with vegetables lower in bitterness and astringency, confirming that females were more familiar than males with vegetables lower in bitterness and astringency, irrespective of PROP status. 

#### 3.2.5. Familiarity with Coffee/Tea (FC+ and FC-)

No effect of age, gender, or their interaction was found on the familiarity index with coffee/tea characterized by high or low bitterness and astringency in any model. 

**Figure 3:**
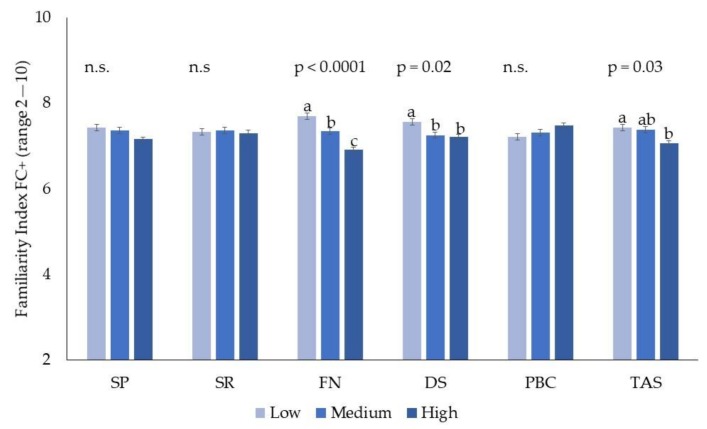
Effect of psychological traits (sensitivity to punishment, SP; sensitivity to reward, SR; food neophobia, FN; sensitivity to disgust, DS; private body consciousness, PBC; and alexithymia, TAS) on the familiarity index with coffee/tea higher in bitterness and astringency (FC+). Different letters represent significant different values (*p* ≤ 0.05).

A significant effect of food neophobia was found on both indices. Neophobic subjects were less familiar with coffee/tea without sugar and more familiar with their version with sugar. Neophilic subjects showed a median familiarity score for this beverage group of eight; this means that, at least occasionally, they consumed both unsweetened coffee and tea or that they regularly consumed only one of these beverages. Neophobic subjects showed a median familiarity value of seven, indicating that they do not consume one of the items and only occasionally consume the other. Individual variations in sensitivity to disgust and alexithymia significantly affected the familiarity index, with coffee/tea characterized by highly unappealing sensations. Subjects with high sensitivity to disgust and high alexithymia were found to be less familiar with the without sugar coffee/tea group of products. The effect of individual variation in psychological traits on the familiarity index for coffee/tea high in bitterness and astringency level is reported in Figure 3. 

No significant effect of PROP was found on either index of familiarity. 

#### 3.2.6. Correlation between Choice of and Familiarity with Bitter/Astringent Option 

Significant positive correlations between the vegetable choice index and familiarity index with vegetables higher in bitterness and astringency, and between the coffee/tea choice index and familiarity index with coffee/tea higher in bitterness and astringency, were found in each subgroup of individuals (low, medium, and high) for each personality trait and in each PROP status class (NT, MT, and ST). The correlation coefficient ranged from 0.25 to 0.41 in the case of vegetables and from 0.42 to 0.57 in the case of beverages (Table 6). 

Individuals lower in food neophobia, sensitivity to punishment, and sensitivity to reward reported significantly lower correlations between the vegetable choice index and familiarity index with vegetables higher in bitterness and astringency compared to individuals higher in these traits. Individuals lower in food neophobia reported a significantly higher correlation coefficient between the coffee/tea choice index/familiarity index with coffee/tea higher in bitterness and astringency compared to individuals higher in food neophobia. The correlation coefficients for the coffee/tea choice index/familiarity index with coffee/tea higher in bitterness and astringency increased in ST compared to NT and MT.

## 4. Discussion

The selection of food and beverages to be included in the CATA questionnaire was performed based on pre-existing sensory data from consumers and trained panels. The vegetable CATA questionnaire included vegetables described by potentially unpleasant sensory properties due to their chemical composition, such as a bitter taste, astringent sensations, objectionable flavours, and a dark, unattractive colour (radicchio, rocket, chicory, asparagus, and radish) [64,65,66,67,68] and vegetables characterized by a sweet taste, delicate flavour, and a bright, appealing colour (pumpkin, lettuce, valerian, green salad, chard, and zucchini) [69,70,71,72]. The range of differences between the two options in each pair was relatively high, with the exception of two pairs (carrot versus cauliflower, and lettuce versus bean sprout), for which these sensory properties were checked by less than 20% of the respondents and a significant difference was found for only one of the two sensory properties. These pairs were included in Study 2 based on the fact that a subtle but significant difference was found for at least one of these sensations (carrot versus cauliflower for bitterness and lettuce versus bean sprout for astringency). The coffee/tea CATA questionnaires included versions of the of the same hot beverage varying in bitter and astringency due to the inclusion or exclusion of ingredients masking the perception of bitterness and astringency (i.e., milk and sweeteners). Findings from the CATA questionnaires confirmed that vegetable and coffee/tea items included in the choice and familiarity indices significantly varied in bitterness and astringency. This substantiates the screening of items based on the hypothesis that they should represent phenol-rich dishes/beverages varying in the level of bitterness and astringency sensations. 

Based on the results from the two CATA questionnaires, it was possible to divide questionnaire items into two groups, each representing the lower and higher bitterness/astringency option for vegetable-based dishes or for coffee/tea beverages, according to consumer expectations. Two main features characterized the approach for food grouping proposed in the present paper: (1) sensory differences between selected vegetable/beverages items were defined according to the response of the target population rather than derived from existing data on other consumer groups (e.g., other food cultures or trained panels); (2) the individual propensity to prefer more or less bitter/astringent options of the phenol-rich foods and beverages was investigated by means of indices computed on choice of and familiarity responses with vegetable and coffee/tea groups rather than considering the response to specific single food/beverage items. These features allowed the highlighting of the importance of individual differences in psychological traits and chemosensory ability in affecting familiarity with, and choice for, phenol-rich foods. The approach based on CATAs to group foods differing in bitter and astringency limits bias due to misinterpretation of the consumer expectation for sensory differences between foods. Furthermore, the computation of indices minimized the impact of individual preferences for specific food/beverages items (for example, a specific bitter vegetable might be very popular and well accepted in some regions and not in others). 

The characteristics of the population participating in the study confirmed existing data on gender and age effects on psychological traits and PROP status. We found no effect of gender on neophobia, in line with previous findings that reported no [73] or small [53] effects, and we confirmed an increase in neophobia with age [46,74,75]. The gender effect for the other traits was also consistent with previous results, with females more sensitive to punishment than males, and males more sensitive to reward than females [54,76], females more sensitive to disgust [36], and no gender effect on alexithymia [59]. For age, with some exceptions, comparisons with previous studies are more limited, considering that much of the extant literature involved younger individuals or a specific age class. In our sample, we found a decrease in alexithymia with age, in contrast to findings in an adult population in Finland [77].

Results from this study confirmed previous findings on the age and gender effect on PROP responsiveness, with aging negatively associated with PROP responsiveness [21,53,78,79,80]. Females rated PROP bitterness higher than males, confirming other results showing that females are more sensitive to PROP than males, and more likely to be tasters [53,80,81]. While females were more familiar with vegetables, independent of their bitterness and astringency, the choice of the most bitter and astringent vegetable option was higher in males than females and increased with aging, irrespective of their psychological traits. A higher preference for sweetness in females is well documented [82] and this may explain our results in the choice test. 

The comparison of choice and familiarity indices for vegetables indicated that bitterness and astringency did not represent a barrier to vegetable consumption in females. At the same time, the choice for bitter/astringent food did not appear a reliable predictor of vegetable consumption in males. A greater appreciation of health-related food aspects, greater nutritional and culinary knowledge, and an increased interest in preparing home-cooked meals are all positively associated with vegetable consumption [83] and were likely to be responsible for the higher familiarity for vegetables in females than in males in the current study. 

The positive association of aging with the choice of vegetables higher in bitterness and astringency can be explained by the repeated exposure—an effect that may allow initial avoidance to be overcome, at least partly through “learned safety” [84]. Thus, a food that is initially disliked could become familiar and potentially preferred [85,86]. Furthermore, the increased attention to the health-related aspects of eating associated with aging [53,87] might further help in promoting choices for healthier vegetable options, even if they are less palatable initially. 

Neither choice of nor familiarity with vegetables was affected by PROP status, consistent with the results of previous study showing a lack of association of bitter vegetable preference with responsiveness to PROP bitterness [24,26,34]. Evidence from recent studies highlighted that a complex network of both genetic and environmental factors appears to influence responsiveness to PROP [18,21]. However, this phenotype is still widely used, with the purpose of exploring the associations of chemosensory ability and vegetable preferences [24,26,88]. Among the several alternative methods for evaluating PROP and determining group assignment, in this study we opted for the one solution test [60,89] and the a priori cut-offs for non-tasters (from 0 to 17), medium tasters (MT from 18 to 52), and supertasters (from 53 to 100) [19,20], widely documented in the literature. Alternative chemosensory indices taking into account broader differences in taste systems might offer a new perspective in looking at the association of dietary style and taste responsiveness phenotypes [90,91]. However, based on the results from the present study, and in line with the newer multidimensional models of food preference and choice, environmental factors might mitigate the impact of biology in determining food preferences, such that phenotype differences in responsiveness to bitterness may not be enough to influence food choice and intake [92]. 

In general, data on choice of and familiarity with vegetables indicated the relevant roles of food neophobia, sensitivity to punishment, and sensitivity to disgust as determinants of vegetable eating. These psychological traits were negatively associated with both the choice of vegetables with higher bitterness and astringency and the familiarity with vegetables in general, irrespective of their sensory properties. This is in line with previous findings, which show that food neophobia in adults is associated with a reduced dietary variety, which is most evident in a lower acceptability and intake, particularly of vegetables, fruits, and protein foods [31,93]. Our findings align also with the hypothesis that higher punishment sensitivity is associated with more unhealthy behaviours, as it was found previously to be associated with a higher sugar intake [43]. Individuals with higher alexithymia declared a lower familiarity with vegetables independently of their bitterness and astringency, while no effect on choice was reported. Similarly, Robino and colleagues [45] reported a negative relationship between alexithymia and stated liking for vegetables. The fact that we did not find an effect of this trait on choice may suggest that this trait modulates vegetable consumption independently from the sensory characteristics of vegetables and thus affects the whole product category.

The correlation between choice and familiarity indices significantly varied according to the level of food neophobia and sensitivity to punishment, thus indicating potential differences between what individuals would like to choose and what they declare they consume normally. The correlation value decreased with neophobia and sensitivity to punishment, indicating that low food neophobia and sensitivity to punishment individuals were likely to have a wider vegetable repertoire. In older adults, a positive association between the willingness to try new foods and a wider variety of consumed vegetables has already been observed [94]. On the other hand, the high level of food neophobia and sensitivity to punishment traits were associated with an increased correlation between choice and familiarity. Neophobic individuals tended to be more consistent with what they preferred and what they declared to consume, and this possibly indicates a restricted spectrum of vegetables included in their daily diet. 

These findings, taken together, confirm the hypothesis that personality variables—specifically food neophobia, sensitivity to punishment, and sensitivity to disgust—may act to facilitate or inhibit the preference and intake of vegetables characterized by unpleasant sensations, consistent with what has been previously found for pungency [35] and, in the case of food neophobia, for bitterness and astringency [34].

Aging was positively associated with the choice of the more bitter/astringent coffee/tea options, suggesting the effects over time of learned positive flavour–flavour and/or flavour consequence conditioning via the stimulatory impact of caffeine, leading to the bitter taste of coffee/tea becoming acceptable [95,96]. Taste motives are among the main reasons for caffeinated beverages consumption [97] and a bitter taste contributes to the appreciation for caffeinated beverages drinkers [15]. 

PROP status did not affect choice and familiarity with coffee/tea items, thus adding to the negative findings in data on causal relationships between PROP bitterness perception and coffee/tea preference and consumption [17,98]. Several factors other than sensory properties, such as functional motives, health beliefs, tradition, and culture, shape the personal preferences for caffeinated beverages [97]. Recent findings on genetic of bitterness perception indicate an opposite causal relationship between PROP responsiveness and coffee and tea consumption [98]. This possibly further accounts for the lack of significant effect of PROP status on choice and familiarity indices, since they are based on responses to both tea and coffee. However, differences in correlations between choice and familiarity indices indicated that ST, more than MT and NT subjects, tended to consume the most preferred option. This may imply that these subjects, more sensitive than the rest of the population to unappealing sensations, tended to adopt more strictly the consumption conditions that better adapt to their personal preference. 

Food neophobia, sensitivity to punishment, and sensitivity to disgust appeared to act as barriers to the choice of the more bitter/astringent coffee/tea options. High food neophobia and sensitivity to disgust levels were associated with a lower familiarity with the unsweetened version of coffee/tea items and to a higher familiarity with the least bitter/astringent option for neophobic subjects only. A lower preference for coffee has been already reported for individuals higher in neophobia [93].

Food neophobia significantly affected the strength of the correlation between the choice and familiarity indices of the most astringent/bitter coffee/tea options. The correlation value was significantly higher in subjects with lower than with higher food neophobia. Habit, defined as a ritual or a daily routine, was one of the main motivational factors for caffeinated beverages consumption [15], but neophobic subjects were less familiar with coffee/tea and were only occasional consumers of unsweetened coffee/tea beverages, and this could account for the weaker correlation between choice and familiarity for unsweetened coffee/tea indices. It has been shown that a variety of motivations play a role in the consumption of coffee beverages [99] and that sensory properties are more relevant for individuals who consume more coffee daily and with a faster caffeine metabolism index [100]. We may hypothesise, therefore, that while for individuals lower in neophobia the sensory properties are of importance, thus explaining their preference for the unsweetened options, for those higher in neophobia, coffee preference may be more explained by situational and social factors (e.g., social rituals). 

While this study benefits from a large sample and the study of the impact of psychological traits on choice, some aspects have remained underexplored. Thus, the foods and beverages considered in the study might differ for properties other than bitterness and astringency, such as texture or energy content. Differences in these aspects might have a role in choice and familiarity that has not been taken into account in the present paper, thus possibly limiting the interpretation of the results. Further studies are encouraged, taking into account a larger variety of dimensions.

## 5. Conclusions

The approach proposed in this study for product grouping based on sensory properties was effective and allowed the investigation of the role of individual differences in chemosensory perception and psychological traits as modulators of phenol-rich foods preference and consumption. Individual differences in psychological traits (food neophobia, sensitivity to punishment, and sensitivity to disgust), rather than responsiveness to PROP, influenced both the preference and consumption of phenol-rich foods. Furthermore, psychological traits significantly affected the degree of coherence between what individuals preferred and what they consumed in their daily life, thus, in the ultimate analysis, determining their diet variety. 

A positive correlation between familiarity and choice was confirmed, but the two measures were found to provide different information. While in vegetables the traits food neophobia, sensitivity to punishment, and sensitivity to disgust were found to be associated with a lower familiarity with vegetables independent of their sensory properties, in coffee/tea, food neophobia, sensitivity to disgust, and alexithymia were associated with a lower familiarity with the unsweetened options. To build on these interpretations of food preference and consumption behaviour, the systematic explorations of individual differences in psychological traits should also take place in applied settings. 

## Figures and Tables

**Table 1:** Pairs of food items included in the vegetable choice questionnaire (V-IT-FCQ) and coffee/tea choice questionnaire (C-IT-FCQ).

Vegetable Choice Questionnaire (V-IT-FCQ).	
**0: Options lower in bitterness and astringency**	**1: Options higher in bitterness and astringency**
Pumpkin risotto	Risotto with radicchio
Lettuce and valerian salad	Radicchio and rocket salad
Green salad	Bean sprout salad
Chard	Chicory
Zucchini	Asparagus
Carrots	Cauliflower
Cucumber	Radish
Coffee/Tea Choice Questionnaire (C-IT-FCQ)	
Macchiato	Coffee
Coffee with sugar	Coffee without sugar
Cappuccino	Coffee
Tea with sugar	Tea without sugar

**Table 2:** Percentage of participants who selected the terms “bitterness” and “astringency” in the check-all-that-apply (CATA) experiment. Cochran’s Q test was used to determine significant differences between samples.

Vegetable Choice Questionnaire (V-IT-FCQ)							
**Option 0 (lower in bitterness and astringency)**	**Option 1 (higher in bitterness and astringency)**		**Bitterness (%)**			**Astringency (%)**	
		***p***	**option 0**	**option 1**	***p***	**option 0**	**option 1**
Pumpkin risotto	Risotto with radicchio	**	1.6	69.9	**	7.1	21.9
Lettuce and valerian salad	Radicchio and rocket salad	**	18.9	82.1	**	6.5	27.9
Green salad	Bean sprout salad		16.4	12.9	*	6.0	13.4
Chard	Chicory	**	27.4	81.6	**	13.4	30.3
Zucchini	Asparagus	**	11.9	34.8	**	5.0	13.4
Carrots	Cauliflower	**	3.0	16.9		7.5	7.0
Cucumber	Radish	**	31.3	46.3	*	19.4	29.9
Coffee/Tea Choice Questionnaire (C-IT-FCQ)							
**Option 0 (lower in bitterness and astringency)**	**Option 1 (higher in bitterness and astringency)**		**Bitterness (%)**			**Astringency (%)**	
		***p***	**option 0**	**option 1**	***p***	**option 0**	**option 1**
Macchiato	Coffee	*	50.5	97.9	*	13.3	41.0
Coffee with sugar	Coffee without sugar	*	19.7	97.9	*	20.2	41.0
Cappuccino	Coffee	*	21.8	97.9	*	6.4	41.0
Tea with sugar	Tea without sugar	*	4.3	67.0	*	30.3	44.1

* *p* ≤ 0.01, ** *p* ≤ 0.001.

**Table 3:** Psychological traits: internal reliability (Cronbach’s α–α), limits of the first (1st Q) and the third (3rd Q) quartiles, number of observations for each group (Low, Medium, High).

Trait	α	1st Q	3rd Q	*n* Low	*n* Medium	*n* High
Sensitivity to Punishment	0.85	5	13	310	537	353
Sensitivity to Reward	0.77	3	9	329	540	331
Food Neophobia	0.86	18	36	334	558	308
Sensitivity to Disgust	0.70	25	33	303	533	364
Private Body Consciousness	0.71	16	21	368	490	334
Alexithymia	0.82	38	55	314	567	312

**Table 4:** Two-way ANOVA: gender, age and their interaction effect on psychological traits and on propylthiouracil (PROP) bitterness scores. F, *p*, and mean values.

Trait	Gender				Age					Gender × Age	
	F	*p*-Value	Mean Values		F	*p*-Value	Mean Values			F	*p*-value
			Females	Males			18–30	31–45	46–60		
Sensitivity to Punishment	37.1	<0.0001	9.9	8.0	32.4	<0.0001	10.5 (a)	8.2 (b)	8.2 (b)	1.6	0.2058
Sensitivity to Reward	72.7	<0.0001	5.1	6.8	85.8	<0.0001	7.6 (a)	5.6 (b)	4.7 (c)	0.8	0.4343
Food Neophobia	0.5	0.4701	27.2	27.7	10.0	<0.0001	26.1 (b)	26.6 (b)	29.7 (a)	0.2	0.8198
Sensitivity to Disgust	90.1	<0.0001	30.6	27.6	14.6	<0.0001	28.0 (b)	29.2 (a)	30.1 (a)	3.0	0.0513
Private Body Consciousness	25.3	<0.0001	18.7	17.4	1.1	0.3410	18.2	18.1	17.7	7.2	0.0008
Alexithymia	0.1	0.7899	46.0	46.2	37.9	<0.0001	49.8 (a)	43.4 (b)	45.0 (b)	0.4	0.6821
PROP	22.8	<0.0001	44.6	36.9	12.6	<0.0001	45.2 (a)	41.3 (a)	35.6 (b)	3.0	0.0495

Different letters indicate significantly different values (*p* ≤ 0.05).

**Table 5:** Three-way ANOVA. Psychological trait level (high, medium, and low), PROP Status (NT, MT, ST), gender, age, and relevant two-way interaction effects on the choice index for vegetables (CV), choice index for coffee/tea (CC), indices for familiarity with vegetables with high (FV+) and low (FV-) bitterness and astringency and indices for familiarity with coffee/tea with high (FC+) and low (FC-) bitterness and astringency. F and *p* values. Significant differences (*p* ≤ 0.05) are emboldened.

	Choice Index for Vegetables		Choice Index for Coffee/Tea		Familiarity with Vegetables Higher in Bitterness and Astringency		Familiarity with Vegetables Lower in Bitterness and Astringency		Familiarity with Coffee/Tea Higher in Bitterness and Astringency		Familiarity with Coffee/Tea Lower in Bitterness and Astringency	
	F	*p*	F	*p*	F	*p*	F	*p*	F	*p*	F	*p*
Sensitivity to Punishment	6.4	**0.0017**	3.4	**0.0323**	11.5	**<0.0001**	4.4	**0.0122**	2.1	0.1259	1.6	0.2055
Gender	21.2	**<0.0001**	0.6	0.4306	9.3	**0.0024**	64.7	**<0.0001**	0.3	0.5740	0.0	0.9520
Age	33.0	**<0.0001**	2.2	0.1085	31.4	**<0.0001**	10.8	**<0.0001**	0.0	0.9862	0.6	0.5285
Gender × SP	0.0	0.9683	2.0	0.1414	0.3	0.7644	0.5	0.6182	2.8	0.0628	0.8	0.4683
Age × SP	1.8	0.1286	0.8	0.5416	1.0	0.4138	1.7	0.1581	0.5	0.7620	0.5	0.7682
Sensitivity to Reward	0.8	0.4392	1.3	0.2696	0.1	0.9507	0.1	0.9164	0.1	0.9186	0.1	0.9351
Gender	25.4	**<0.0001**	0.4	0.5273	4.4	**0.0369**	56.3	**<0.0001**	0.8	0.3789	0.0	0.8636
Age	36.2	**<0.0001**	1.8	0.1607	37.8	**<0.0001**	12.6	**<0.0001**	0.2	0.8098	0.1	0.9339
Gender × SR	1.7	0.1766	0.6	0.5717	0.5	0.6215	0.3	0.7440	1.5	0.2328	0.3	0.7591
Age × SR	0.2	0.9501	0.6	0.6883	1.0	0.4203	0.2	0.9312	1.4	0.2288	0.4	0.7848
Food Neophobia	11.7	**<0.0001**	6.8	**0.0012**	34.1	**<0.0001**	14.9	**<0.0001**	16.1	**<0.0001**	5.4	**0.0048**
Gender	32.0	**<0.0001**	0.2	0.6378	3.6	0.0595	58.5	**<0.0001**	0.1	0.7986	0.0	0.8339
Age	40.0	**<0.0001**	4.2	**0.0159**	47.9	**<0.0001**	18.3	**<0.0001**	0.7	0.5207	0.3	0.7172
Gender × FN	1.5	0.2130	0.4	0.6563	0.8	0.4484	1.3	0.2825	1.1	0.3275	0.2	0.8262
Age × FN	0.2	0.9313	2.0	0.0967	1.0	0.4138	0.8	0.5048	0.9	0.4711	0.7	0.5971
Sensitivity to Disgust	13.0	**<0.0001**	4.2	**0.0154**	10.1	**<0.0001**	2.9	0.0545	3.8	**0.0233**	2.2	0.1071
Gender	14.4	**0.0002**	0.8	0.3572	9.6	**0.0019**	58.8	**<0.0001**	2.9	0.0894	0.1	0.7851
Age	45.7	**<0.0001**	3.9	**0.0201**	49.6	**<0.0001**	16.9	**<0.0001**	0.6	0.5310	0.5	0.6163
Gender × DS	0.2	0.7832	0.3	0.7663	0.2	0.8558	0.7	0.4758	1.3	0.2706	0.9	0.4071
Age × DS	0.7	0.6250	1.4	0.2469	1.8	0.1198	1.0	0.3823	1.4	0.2297	1.1	0.3743
Private Body Consc.	0.9	0.4203	0.0	0.9670	4.4	**0.0123**	1.7	0.1773	2.0	0.1346	1.2	0.2918
Gender	24.4	**<0.0001**	0.4	0.5240	3.9	**0.0489**	49.3	**<0.0001**	0.3	0.5837	0.0	0.8951
Age	40.1	**<0.0001**	2.4	0.0889	42.9	**<0.0001**	15.5	**<0.0001**	0.7	0.4892	0.4	0.6871
Gender × PBC	3.6	**0.0267**	0.2	0.8372	2.2	0.1113	0.4	0.7034	0.2	0.8094	1.7	0.1922
Age × PBC	2.0	0.0905	1.5	0.1919	2.3	0.0603	0.8	0.5297	1.3	0.2852	0.7	0.6041
Alexithymia	2.1	0.1184	2.9	0.0547	7.7	**0.0005**	5.4	**0.0046**	3.5	**0.0292**	1.5	0.2127
Gender	20.8	**<0.0001**	1.2	0.2750	5.5	**0.0195**	56.3	**<0.0001**	0.7	0.4148	0.2	0.6722
Age	30.5	**<0.0001**	2.0	0.1400	32.2	**<0.0001**	10.0	**<0.0001**	0.0	0.9958	0.4	0.6632
Gender × TAS	0.8	0.4407	3.0	**0.0504**	0.4	0.6933	0.0	0.9542	2.0	0.1423	1.4	0.2358
Age × TAS	1.3	0.2528	1.2	0.3312	0.1	0.9693	0.3	0.8856	0.6	0.6304	0.3	0.8903
PROP	0.5	0.5969	0.6	0.5439	0.1	0.8819	0.0	0.9585	0.3	0.7432	1.5	0.2324
Gender	25.7	**<0.0001**	0.8	0.3615	7.4	**0.0067**	67.0	**<0.0001**	1.1	0.2856	0.0	0.9142
Age	33.2	**<0.0001**	2.5	0.0848	39.2	**<0.0001**	14.1	**<0.0001**	0.4	0.6744	0.4	0.6583
Gender × PROP	1.2	0.2968	0.2	0.8411	3.0	0.0526	5.5	**0.0042**	1.8	0.1711	0.0	0.9752
Age × PROP	0.9	0.4888	0.5	0.7087	0.7	0.6255	0.2	0.9591	0.3	0.8853	1.7	0.1386

SP: Sensitivity to punishment; SR: Sensitivity to reward; FN: Food neophobia; DS: Sensitivity to disgust; PBC: Private Body consciousness; TAS: Alexithymia; PROP: PROP taster status.

**Table 6:** Pearson correlation coefficients between the vegetable choice index (CV) and familiarity index with vegetables higher in bitterness and astringency (FV+) and the Pearson correlation coefficients between the coffee/tea choice index (CC) and familiarity index with coffee/tea higher in bitterness and astringency (FC+) within the three levels (low, medium, high) of each psychological trait and PROP status (NT, MT, ST).

Vegetable Choice Index/Familiarity Index with Vegetables Higher in Bitterness and Astringency (CV/FV+)				
**Trait**	**Low**	**Medium**	**High**	**Diff. among groups**
Sensitivity to Punishment	0.25	0.38	0.38	*
Sensitivity to Reward	0.28	0.40	0.37	*
Food Neophobia	0.25	0.37	0.41	*
Sensitivity to Disgust	0.34	0.39	0.32	n.s.
Private Body Consciousness	0.34	0.41	0.32	n.s.
Alexithymia	0.33	0.36	0.37	n.s.
**PROP status**	**NT**	**MT**	**ST**	
PROP	0.30	0.38	0.37	n.s.
Coffee/tea choice index/familiarity index with coffee/tea higher in bitterness and astringency (CC/FC+)				
**Trait**	**Low**	**Medium**	**High**	**Diff. among groups**
Sensitivity to Punishment	0.49	0.50	0.56	n.s.
Sensitivity to Reward	0.56	0.51	0.49	n.s.
Food Neophobia	0.57	0.53	0.42	*
Sensitivity to Disgust	0.55	0.51	0.50	n.s.
Private Body Consciousness	0.54	0.49	0.54	n.s.
Alexithymia	0.55	0.52	0.48	n.s.
**PROP status**	**NT**	**MT**	**ST**	
PROP	0.49	0.49	0.57	*

All correlations are significant (*p* ≤ 0.05). * significant pairwise differences. Vegetables—Sensitivity to Punishment: Low–Medium (*p* = 0.02), Low–High (*p* = 0.03); Sensitivity to Reward: Low–Medium (*p* = 0.03); Food Neophobia: Low–Medium (*p* = 0.03), Low–High (*p* = 0.01). Coffee/tea—Food Neophobia Low–High (*p* = 0.01), Medium–High (*p* = 0.02), PROP status: Medium–High (*p* = 0.05). n.s. = non-significant (*p* > 0.05).

## Abbreviations

DS: Sensitivity to Core Disgust; FN: Food Neophobia; PBC: Private Body Consciousness; SP: Sensitivity to Punishment; SR: Sensitivity to Reward; TAS: Alexithymia; V-IT-FCQ: Vegetable Choice Questionnaire; C-IT-FCQ: Coffee/Tea Choice Questionnaire; CV: Vegetable Choice Index; CC: Coffee/Tea Choice Index; FV+/FV-: Indices of Familiarity with vegetables high (+) or low (-) in bitterness and astringency sensations; FC+/FC-: Indices of Familiarity with coffee/tea high (+) or low (-) in bitterness and astringency sensations
